# Supplementary material for: Phytochemical Analysis, Biological Activities, and Docking of Phenolics from Shoot Cultures of Hypericum perforatum L. Transformed by Agrobacterium rhizogenes
Source: Molecules. 2024 Aug 17;29(16):3893. doi: 10.3390/molecules29163893 (PMC11357161; doi:10.3390/molecules29163893)
Supplement: Supplementary file 1 [file molecules-29-03893-s001.zip › molecules-3136894-supplementary.pdf]

Supplementary Material

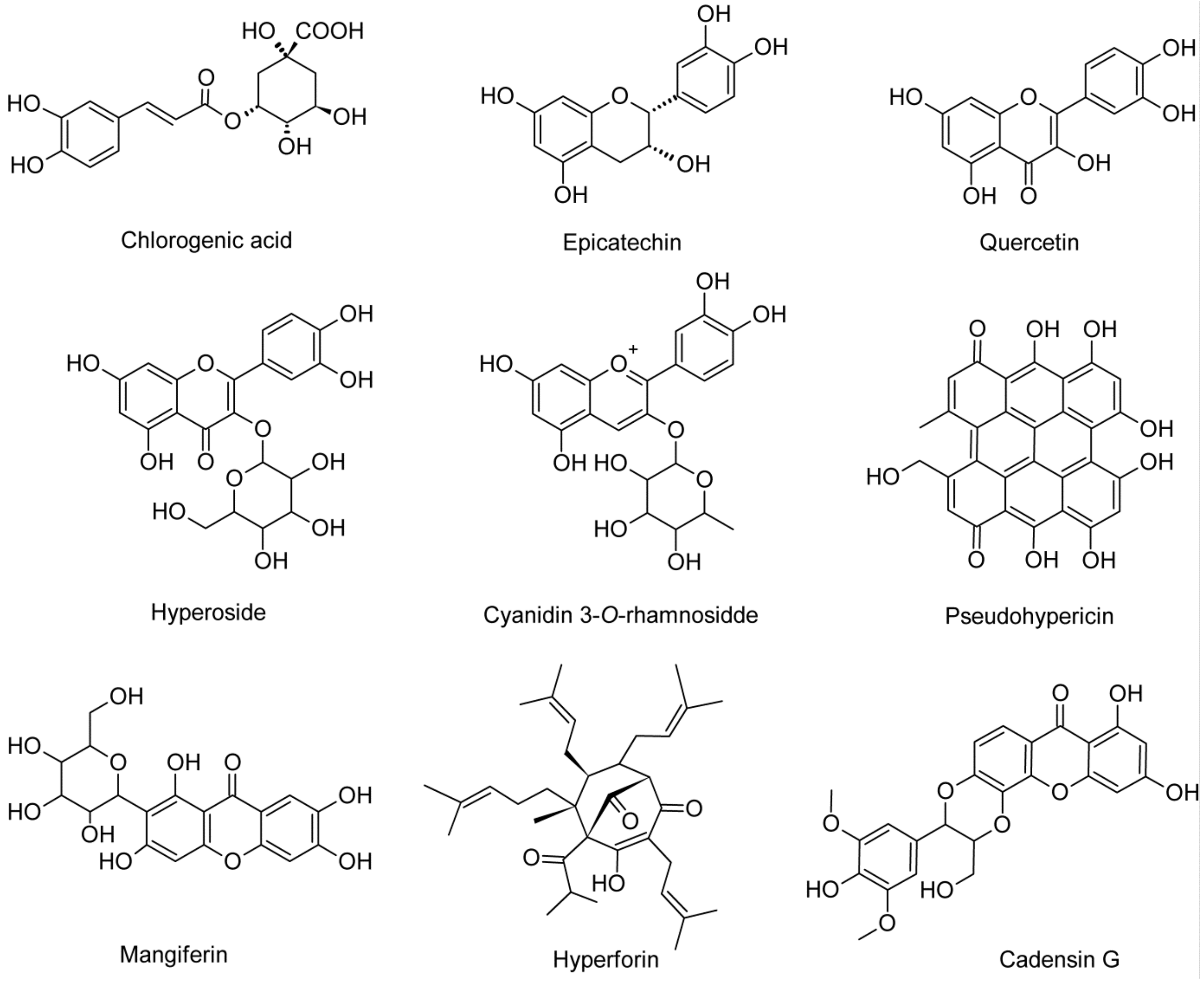

Figure S1. The structures of the most abundant phenolic compounds in *Hypericum perforatum* shoot cultures.

Table S1. Binding energy and inhibition constant of the best-ranked docking pose of other tested ligands and enzymes.*

Ligands	Enzymes	Binding energy ($kcal \cdot mol^{-1}$)	Inhibition constant (K_i)
Quercitrin	MAO-A	-4.41	581.45 μM
	AChE	-7.98	1.41 μM
	BChE	-8.34	770.86 nM
	TYR	-4.68	372.90 μM
	α-AM	-6.64	13.53 μM
	α-GL	-6.92	8.53 μM
	PL	-9.58	94.00 nM
	CHE	-5.40	110.45 μM
Hyperoside	MAO-A	-6.85	9.52 μM
	AChE	-8.37	735.63 nM
	BChE	-8.55	544.39 nM
	TYR	-3.73	1.84 mM
	α-AM	-5.64	73.00 μM
	α-GL	-6.33	22.92 μM
	PL	-8.76	377.62 nM
	CHE	-5.90	47.33 μM
Cyanidin 3-*O*-rhamnoside	MAO-A	-4.67	377.41 μM
	AChE	-7.96	1.46 μM
	BChE	-8.91	259.09 nM
	TYR	-4.80	302.32 μM
	α-AM	-6.60	14.5 μM
	α-GL	-7.97	1.45 μM
	PL	-8.24	917.80 nM
	CHE	-6.28	24.95 μM
Mangiferin	MAO-A	-5.94	43.98 μM
	AChE	-7.86	1.74 μM
	BChE	-6.59	14.74 μM
	TYR	-3.72	1.88 mM
	α-AM	-5.32	126.49 μM
	α-GL	-7.81	1.90 μM
	PL	-7.59	2.75 μM
	CHE	-5.35	119.40 μM
Cadensin G	MAO-A	-8.18	1.01 μM
	AChE	-10.62	16.49 nM
	BChE	-8.27	860.00 nM
	TYR	-5.49	93.90 μM
	α-AM	-6.12	32.42 μM
	α-GL	-8.13	1.09 μM
	PL	-8.40	697.99 nM
	CHE	-8.38	723.46 nM

* MAO-A: monoamine oxidase-A; AChE: acetylcholinesterase; BChE: butytylcholinesterase; TYR: tyrosinase; α-AM: α-amylase; α-GL: α-glucosidase; PL: pancreatic lipase; CHE: cholesterol esterase.

Table S2. Retention times, UV and mass spectral data of phenolic compounds in *Hypericum perforatum* shoot extracts.*

Peak	Phenolic compounds	t_R (min)	UV (nm)	$[M–H]^-$ (*m/z*)	MS^2 $[M–H]^-$ (*m/z*)
**Phenolic acids**					
F2	Chlorogenic acid	18.83	240, 294, 326	353	**191**, 179, 135
F3	3-*p*-Coumaroylquinic acid	25.09	314	337	191, **163**
F5	3-Feruloylquinic acid	29.11	314	367	193
**Flavan-3-ols**					
F1	(epi)catechin-(epi)gallocatechin dimer	18.10	254, 296	593	425, 407, **289**
F4	Procyanidin B2	27.89	280	577	559, 451, **425**, 407, 289
F6	Procyanidin trimer	31.08	280	865	739, **695**, 577
F7	(epi)catechin	32.02	280	289	**245**, 205
**Flavonol glycosides and aglycons**					
F9	Quercetin 6-*C*-glucoside	36.26	252, 284, 326	421	331, **301**, 258
F11	Kaempferol 6-*C*-glucoside	38.13	256, 266, 350	405	315, **285**
F12	Hyperoside (quercetin 3-*O*-galactoside)	41.58	256, 356	463	**301**
F13	Rutin (quercetin 3-*O*-rutinoside)	44.20	263, 298sh, 356	609	**301**
F14	Quercitrin (quercetin 3-*O*-rhamnoside)	47.86	254, 354	447	**301**
F15	Quercetin	54.70	256, 372	301	**179**, 151
**Anthocyanins**					
F8	Cyanidin 3-*O*-glycoside	33.56	298, 520	449 $[M+H]^+$	**287**
F10	Cyanidin 3-*O*-rhamnoside	37.16	234, 280, 522	433 $[M+H]^+$	**287**
**Naphthodianthrones**[a]					
F16	Pseudohypericin	81.70	234, 286, 326, 544, 590	519	487, 421
F17	Hypericin	84.20	288, 325, 465, 590	503	**405**
F18	Protopseudohypericin	85.90	218, 254, 368, 542	521	477, **423**, 385, 317
**Acyl-phloroglucinols**					
F19	Hyperforin	86.06	204, 224, 278	535	467, **383**, 315, 271
F20	Adhyperforin	86.49	206, 280	549	**397**
**Xanthones**					
X1	Mangiferin	33.92	238, 256, 318, 366	421	**331**, **301**, 258
X2	Brasilixanthone B	37.93	254, 289, 330	391	377, 359
X3	Trihydroxyxanthone-sulfonate	38.97	232, 258, 374	323	**309**, 243
X4	Dimethylmangiferin	41.03	258, 319, 364	449	315, **301**, 285
X5	Dihydroxy-metoxyxanthone-sulfonate	52.62	250, 312, 356	337	321, 257
X6	Mangiferin *C*-prenyl isomer	62.23	238, 260, 312, 372	489	399, **327**
X7	1,3,6,7-Tetrahydroxyxanthone 2-prenyl xanthone	75.54	248, 312, 368	327	311, 283, **271**, 257
X8	1,3,6,7-Tetrahydroxyxanthone 8-prenyl xanthone	76.65	248, 312, 368	327	325, **297**, 258, 201
X9	γ-Mangostin	81.11	246, 262, 320	395	339, 297, **283**, 271
X10	5-*O*-Methyl-2-deprenylrheediaxanthone B	82.60	252, 286, 330	331	262, 234, 193
X11	Cadensin G	84.16	270, 330, 400	467	257, 227

[*] MS^2 ions in bold indicate the base peak. sh: shoulder; t_R: retention time.
